# Antioxidant System Response and cDNA-SCoT Marker Profiling in *Phoenix dactylifera* L. Plant under Salinity Stress

**DOI:** 10.1155/2017/1537538

**Published:** 2017-06-18

**Authors:** Fahad Al-Qurainy, Salim Khan, Mohammad Nadeem, Mohamed Tarroum, Abdel-Rhman Z. Gaafar

**Affiliations:** Department of Botany and Microbiology, College of Science, King Saud University, Riyadh 11451, Saudi Arabia

## Abstract

Many *Phoenix dactylifera* (date palm) cultivars are grown in the arid and semiarid regions of the world, including Saudi Arabia. *P. dactylifera* is highly tolerant to salinity stress. To investigate the response of Khalas cultivar of *P. dactylifera*, two-month-old plants were treated with sodium chloride (50, 100, and 150 mM NaCl) for three months. Our result showed that proline content was higher in all treated plants compared to control plants. Thiobarbituric acid reactive substances (TBARS) were increased at 100 and 150 mM NaCl treatments; however, the result was found nonsignificant between control and plants treated at 50 mM NaCl. Similarly, enzyme activities of catalase (CAT) and superoxide dismutase (SOD) were 0.805 and 0.722 U/mg protein/min, respectively, and were greater at 100 and 150 mM NaCl treatments compared to the control plants. Total chlorophyll content and fresh weight of shoots and roots decreased substantially with the increase of salinity. A cDNA start codon-targeted (cDNA-SCoT) marker showed a variation in different gene expressions profiling between treated and untreated plants under various NaCl concentrations.

## 1. Introduction

In recent decades, soil salinity has become a global agricultural constraint [1, 2]. Salinity is increasing on Arabic land, and more than 50% would be salinized by the year 2050, if suitable corrections are not made [3]. Furthermore, the salinized areas are increasing every year at a rate of 10% for different reasons including poor cultural practices, irrigation with saline water, weathering of native rocks, high surface evaporation, and low precipitation [4, 5]. Salt stress causes average yield losses of more than 50% in major crops in agriculture-based countries [6]. Reactive oxygen species (ROS) are produced in plant cells under salinity stress [7], which can damage the cells. It also affects many metabolic and physical processes of the plant, and as a result, the growth is hampered [8]. A high salinity stress causes osmotic and ionic stresses in the plant cells, which lead to several physiological and morphological modifications [9].

*Phoenix dactylifera* (date palm) is the main horticultural fruit tree in many arid and semiarid countries in the Middle East, North Africa, and Central America [10]. *P. dactylifera* can survive under extreme abiotic stresses, including conditions of drought, high temperature, and relatively high soil salinity levels [11–14]. The salinity stress affected the large area of arid and semiarid regions of agricultural field [15] and has impacted more losses in *P. dactylifera* and other crop species [16].

The antioxidant enzyme activities such as catalase (CAT) and superoxide dismutase (SOD) increase under salinity stress for scavenging regenerated ROS to protect the cell from damage [17, 18]. The enzyme SOD is found in various compartments of the cell and catalyzes the superoxide radicals (O_2_^−^) to H_2_O_2_ and O_2_ [19]. The H_2_O_2_ is removed from the cell by peroxidases and catalase [19–22].

The proline, an osmoprotectant, is produced under abiotic and biotic stresses [23] in plants. Heat and cold treatments can result in a significant increase in proline level in the leaves and roots of *P. dactylifera* [14]. The changes occurred in SOD and chlorophyll a/b-binding protein under salt stress in *P. dactylifera* [24]. Thiobarbituric acid reactive substances (TBARS), which are produced in the plant cells, act as a potential indicator of damage under induction of stresses [25]. An increase in TBARS content under salinity stress can cause damage to membranes and also to particular cell tissues [26–28]. Usually, osmotic or salt stress induces TBARS accumulation [29]. TBARS accumulation in cowpea leaves under salinity stress depends on exposure time [30]. However, a reduction in TBARS level under salinity stress is poorly reported in the literature [31].

Different methods have been developed for the gene expression study in plants or animals such as cDNA microarray, cDNA-SRAP, cDNA-AFLP, serial analysis of gene expression (SAGE), suppression subtractive hybridization (SSH), representational difference analysis (RDA), and mRNA differential display (DD) [32–42]. All these markers have advantages and disadvantages based on the reproducibility of the results, available resources, technical expertise, and cost of development.

A cDNA start codon-targeted (cDNA-SCoT) marker has been used for the study of gene expression in *Saccharum officinarum*, *Mangifera indica*, *Phoenix dactylifera*, and *Dendrobium officinale* [43–46]. However, this marker has also been used for the assessment of genetic diversity in various plant species [47–50]. A high degree of variability has been found among the germplasms of *P. dactylifera* under salinity and drought stresses [51]. Knowledge of molecular mechanisms under salinity and drought conditions in *P. dactylifera* is limited [52–57]. In the present study, we performed experiments on the Khalas cultivar of *P. dactylifera* to determine the antioxidant system response and gene expression profiling under salinity stress.

## 2. Materials and Methods

A pot experiment was conducted in a growth chamber for salinity stress treatments in four replicates. The pots were filled with a mixture of sand and peat moss (3 : 1). The healthy seeds of *P. dactylifera* were surface sterilized with sodium hypochlorite solution (4.0% available chlorine) for 10 min and washed thoroughly four times with distilled water. The seeds were sown in plastic pots and watered at regular interval to maintain moisture for better germination. Salinity stress treatments were given to the two-month-old plants of Khalas cultivar of *P. dactylifera* for three months. Three concentrations of NaCl as low (T-50, 50 mM), intermediate (T-100, 100 mM), and high (T-150, 150 mM) were used to treat the plants. Each concentration of salt solution (100 ml) was given to each pot after two-week time intervals. 100 ml of 1/4 strength MS solution was added to each pot after two-week time intervals. The pots were maintained in the growth chamber at 26-27°C, photoperiod 16 h per day, and relative humidity of 72%. The salt-treated and untreated plants were harvested after three months. Biochemical and molecular parameters were subsequently taken to study the antioxidant system response of *P. dactylifera* under salinity stress.

### 2.1. Biomass and Morphological Traits

Fresh leaf and root weight and shoot and root length were measured after three months of salinity treatment. Each treatment was compared to control plants for the evaluation of their salt stress responses.

### 2.2. Proline Estimation

The proline was estimated using the method developed by Hanson et al. [58]. Fresh leaves (0.3 g) were ground in 10 ml of aqueous sulphosalicylic acid (3%). The mixture was centrifuged for 15 min at 9000 ×g. The supernatant (2 ml) from the above step was taken and mixed with an equal volume of acid ninhydrin (1.25 g ninhydrin in 30 ml acetic acid and 20 ml of 6 N H_3_PO_4_) and acetic acid. The mixture was placed for 1 h in boiling water for incubation. After incubation, the mixture was taken out from the boiling water and immediately placed in an ice bath. 4 ml of toluene was added in the mixture (4 ml) after taking it from the ice water bath. The mixture was vortexed, and chromatophore-containing toluene was separated from the aqueous phase. The absorbance was taken at 520 nm (Model UB-1800, Shimadzu, Japan) to determine proline content.

### 2.3. Total Chlorophyll

Total chlorophyll was estimated according to the Arnon method [59]. The leaves were separated and washed with DDW; 0.1 g of chopped leaves was placed in the test tubes for each treatment, and 10 ml of DMSO was added to each test tube. The tubes were kept in an oven at 65°C. After 120 minutes, the tubes were taken out and the absorbance of the solution was recorded immediately at 663 nm and 645 nm on a UV-vis spectrophotometer (Model UB-1800, Shimadzu, Japan). The pigment concentration was calculated in *μ*g/ml for treated and untreated samples.

### 2.4. Superoxide Dismutase (SOD)

The activity of superoxide dismutase (EC 1.15.1.1) was measured according to the method developed by Dhindsa et al. [60]. A fresh sample (0.05 g) was homogenized in 2 ml of extraction mixture containing phosphate buffer (0.5 M, pH 7.3), 0.3 mM-EDTA, 1% Triton × 100 (*w*/*v*), and 1% PVP (*w*/*v*). The mixture was centrifuged for 10 min at 4°C at 10,000 ×g. The supernatant was taken after centrifugation for the assay of SOD activity. The assay mixture, consisting of 1.5 ml reaction buffer, 0.2 ml of methionine, 0.1 ml of each (1 M-NaCO_3_, 2.25 mM-NBT solution, 3 mM-EDTA, riboflavin, and enzyme extract), and 1 ml of DDW, was incubated under the light. The blank mixture containing all substances was kept in the dark. Absorbance of samples along with the blank mixture was read at 560 nm using the UV-vis spectrophotometer (Model UB-1800, Shimadzu, Japan). A 50% reduction in color was considered as one enzyme unit (EU). The activity of SOD was calculated in EU (mg^−1^ protein min^−1^).

### 2.5. Catalase (CAT)

The activity of catalase (EC 1.11.1.6) was determined in the leaves using the method of Aebi [61]. 0.5 g of fresh leaf samples was ground in extraction buffer (5 ml) containing phosphate buffer (0.5 M, pH 7.3), 0.3 mM-EDTA, and 0.3 mM-H_2_O_2_. The mixture was centrifuged for 20 min at 10,000 ×g at 4°C. The reaction was carried out in 2 ml of reaction mixture (0.1 ml, 3 mM-EDTA, 0.1 ml of enzyme extract, and 0.1 ml of 3 mM-H_2_O_2_) for 5 min. CAT activity was estimated at 240 nm using the UV-vis spectrophotometer with the help of extinction coefficient (*R*) 0.036 mM^−1^cm^−1^ and expressed in EU (mg^−1^ protein min^−1^).

### 2.6. Thiobarbituric Acid Reactive Substances (TBARS)

TBARS content was determined in the leaves using the method developed by Cakmak and Horst [62] with minor modification. The fresh leaf samples (0.5 g) were ground in 5 ml of 0.1% (*w*/*v*) trichloroacetic acid (TCA). The centrifugation was performed for 5 min at 12,000 ×g for supernatant collection. The supernatant was taken from the above step, and 1 ml of it was added to 4 ml of 0.5% (*w*/*v*) TBA in 20% (*w*/*v*) TCA. The mixture was placed for 30 min at 90°C in water bath, and thereafter, the reaction was terminated in an ice bath. The centrifugation was performed for 5 min at 10,000 ×g for supernatant collection. The absorbance of the supernatant was read at 532 and 600 nm wavelengths on a spectrophotometer (Model UB-1800, Shimadzu, Japan). The TBARS content was calculated using the following formula:

(1) $$\begin{array}{c} TBARS\;\left({nmol\;g}^{-1}\;fw\right)=\frac{\left(A_{532}-A_{600}\right)\times V\times 1000}{155\;\left(extinction\;coefficient\right)\times W}, \end{array}$$

where *A*_532_ = absorbance at 532 nm, *A*_600_ = absorbance at 600 nm, *V* = extraction volume, and *W* = fresh weight of tissue.

### 2.7. RNA Extraction for cDNA-SCoT Marker Profiling

Total RNA was isolated from the control and salinity-stressed plants using the RNeasy plant mini kit (Qiagen) according to the instructions given in the manual. The quantity and quality were measured using the spectrophotometer (Nanodrop 8000, Thermo Scientific). High quality of cDNA was prepared using the QuantiTect Reverse Transcription Kit (Qiagen). The PCR reaction was performed in a total volume of 25 *μ*l using the SCoT primers (Table 1) for the study of expression profiling. These primers were selected from the literature of monocot plant species [63]. The PCR bead (GE Healthcare, UK) was used for PCR amplification. The cDNA was diluted in RNase-free water to working concentration 50 ng for PCR amplification with SCoT primer (20 picomole per reaction). PCR was performed in an AB Veriti 96-well thermal cycler. The cycling profile was 94°C for 3 min, 45 cycles at 94°C for 1 min, 44.5°C for 30 s, 72°C for 1 min, and a cycle of 72°C for 5 min. The amplified products were resolved on 1.3% TBE agarose gel.

### 2.8. Statistical Analysis

The data recorded in all experiments were statistically analyzed by using IBM SPSS STATISTICS 19. Data from each parameter was subjected to a one-way analysis of variance (ANOVA); the post hoc comparison for the observation was assumed by Duncan's test. The data shown are the averages of four replicates and were statistically significant at the *p* < 0.05 level.

## 3. Results and Discussion

Free radicals, or ROS, are produced in plant cells under stress conditions and may react with pigments, lipids, proteins or nucleic acids which leads to membrane damage, lipid peroxidation, and inactivation of enzymes, thus affecting the cell viability [64, 65]. Plant gene expression analysis is very important in agriculture under biotic and abiotic stresses as it promotes genetic improvement of other crops for their yield and quality traits.

**Figure 1:**
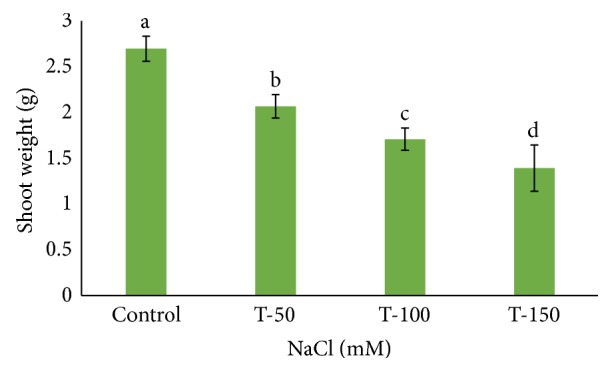
Fresh shoot weight in *Phoenix dactylifera* grown in the pot at different concentrations of NaCl. Data represent means of four replicates ± standard deviation. Different letters on bars represent the significant values according to Duncan's test (*p* < 0.05).

**Figure 2:**
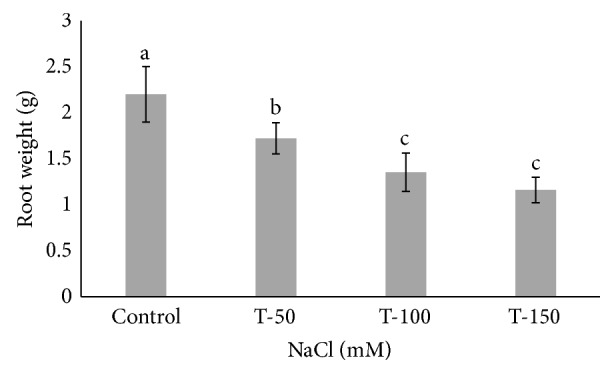
Fresh root weight in *Phoenix dactylifera* grown at different concentrations of NaCl. Data represent means of four replicates ± standard deviation. Different letters on bars represent the significant values according to Duncan's test (*p* < 0.05).

**Figure 3:**
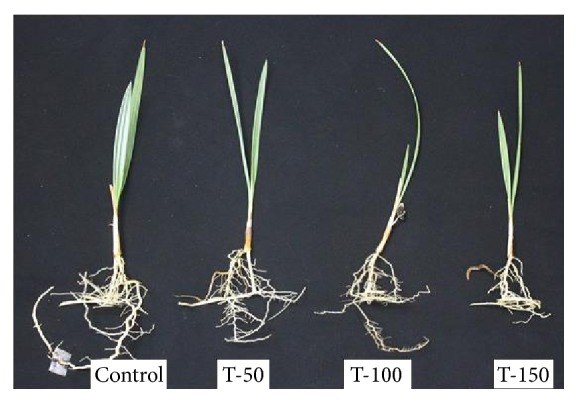
Morphological variations in the root and shoot of *Phoenix dactylifera* grown under different NaCl concentrations (control: 0 mM, T-50: 50 mM, T-100: 100 mM, and T-150: 150 mM) for 3 months.

**Figure 4:**
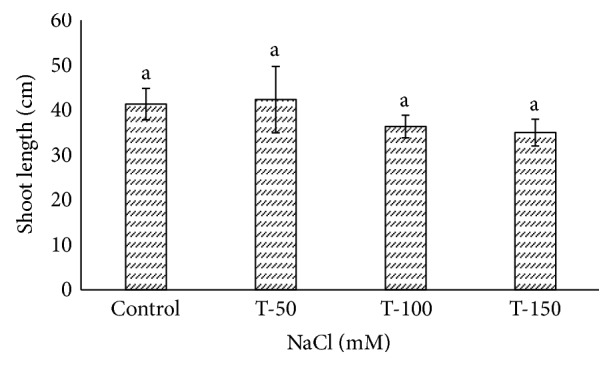
Shoot length variation in *Phoenix dactylifera* grown at different concentrations of NaCl. Data represent means of four replicates ± standard deviation. Different letters on bars represent the significant values according to Duncan's test (*p* < 0.05).

**Figure 5:**
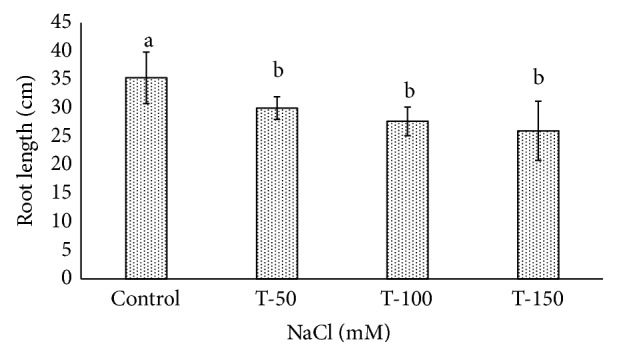
Root length variation in *Phoenix dactylifera* grown at different concentrations of NaCl. Data represent means of four replicates ± standard deviation. Different letters on bars represent the significant values according to Duncan's test (*p* < 0.05).

**Figure 6:**
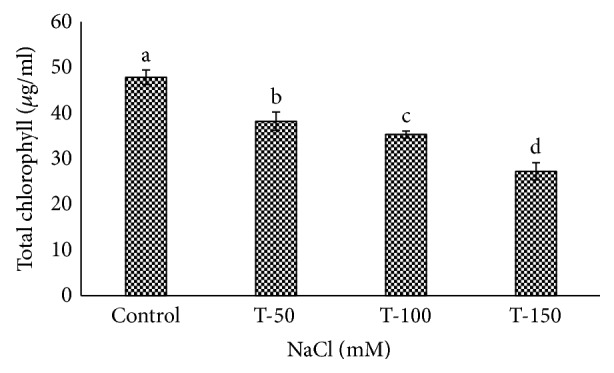
Effect of salinity on chlorophyll content in *Phoenix dactylifera*. Data represent means of four replicates ± standard deviation. Different letters on bars represent the significant values according to Duncan's test (*p* < 0.05).

Fresh weight of shoot and root of *P. dactylifera* decreased significantly as the salinity increased (Figures 1 and 2). High salinity stress caused more reduction in the weight of shoot and root (1.392 and 1.160 g) as compared to control plants (2.697 and 2.201 g), respectively. Alkhateeb et al. [66] performed experiments on *P. dactylifera* under salinity stress and found that growth declined with increasing salinity stress. Excess salinity affects plants severely due to water stress, membrane disorganization, nutritional disorders, ion toxicity, and the expansion and reduction of cell division [67, 68]. The root length was more affected than the shoot length (Figure 3). The more reduction in the root length (26 cm) was observed significantly at 150 mM NaCl when compared to control plants (35.33 cm). The shoot length was less affected under salinity stress, and a nonsignificant variation was found among treated as well as control plants (Figures 4 and 5). There was no effect of salinity observed on the shoot length up to 50 mM NaCl, and the plant grew normally like a normal plant. Ramoliya and Pandey [69] studied on some *P. dactylifera* varieties under salinity stress and found that some varieties can tolerate high levels of soil salinity (12.8 ds m^−1^) without a visible effect. Total chlorophyll content decreased significantly in all treated *P. dactylifera* plants as the salinity increased. A low chlorophyll content (27.241 *μ*g/ml) was observed in plants treated at high salinity stress (Figure 6) compared to control plants (47.873 *μ*g/ml). Similarly, chlorophyll *a* and *b* decreased under salinity stress in a date palm [70].

**Figure 7:**
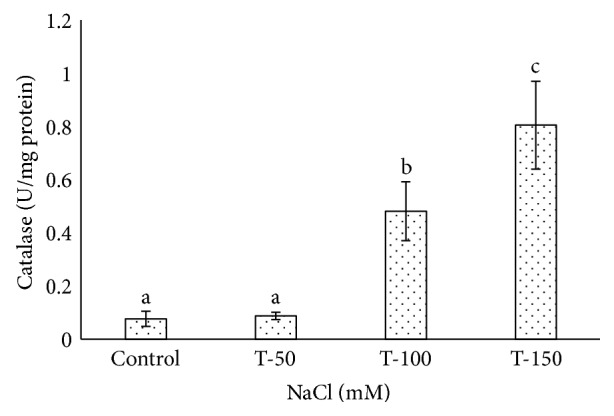
Catalase activity in *Phoenix dactylifera* grown at different concentrations of NaCl. Data represent means of four replicates ± standard deviation. Different letters on bars represent the significant values according to Duncan's test (*p* < 0.05).

**Figure 8:**
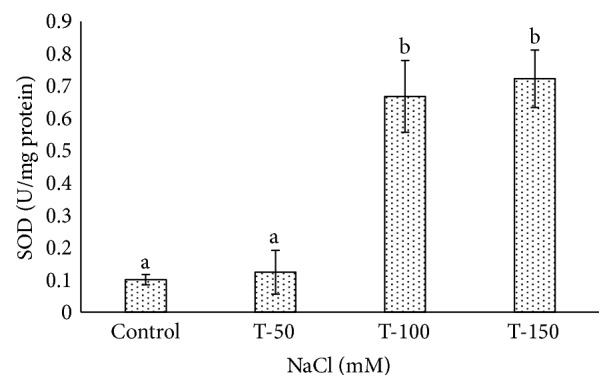
Superoxide dismutase activity in *Phoenix dactylifera* grown at different concentrations of NaCl. Data represent means of four replicates ± standard deviation. Different letters on bars represent the significant values according to Duncan's test (*p* < 0.05).

Catalase activity increased significantly in the leaves of date palm plants under 100 and 150 mM NaCl treatments, and it was 0.481 and 0.805 U/mg protein/min, respectively (Figure 7). However, a very low CAT activity (0.087 U/mg protein/min) was observed at 50 mM NaCl nonsignificantly compared to control plants. CAT activity induced at an application of 100 mM NaCl in wild *Lycopersicon pennellii* [71]. SOD activity increased under 100 mM NaCl and 150 mM NaCl treatments compared to nontreated plants (Figure 8). A very low SOD activity of 0.138 U/mg protein/min was found in plants treated at 50 mM NaCl. A high SOD enzyme activity of 0.667 and 0.722 U/mg protein/min was found significantly at 100 mM NaCl and 150 mM NaCl treatments compared to the control plants, where SOD activity only reached 0.1 U/mg protein/min. Our results were consistent with earlier findings for *P. dactylifera* where catalase and peroxidase activities increased under salinity treatments [70]. The CAT and SOD activities also increased in *P. dactylifera* Hillawi cv [72] under salinity stress. The activities of CAT and SOD were enhanced at 100 mM NaCl in wild *Lycopersicon pennellii* [71]. The responses of SOD and CAT were high under salinity stress (120 and 240 mM NaCl) in the leaves of two-week-old seedlings of barley, and it was found significant [73]. An increase in antioxidant enzymes under stressful conditions plays an important role to overcome the oxidative stress and often correlates to the type and magnitude of the stress [65].

**Figure 9:**
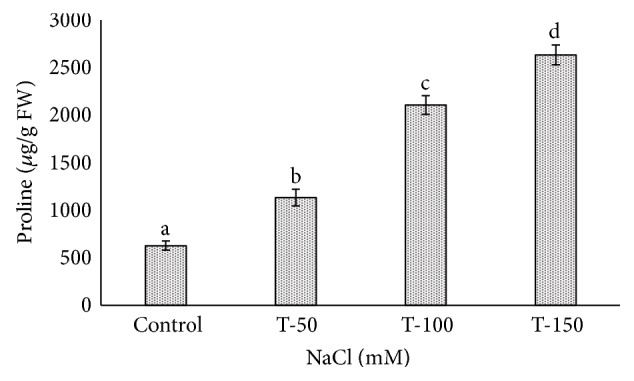
Proline accumulation in the leaf of *Phoenix dactylifera* grown at different concentrations of NaCl. Data represent means of four replicates ± standard deviation. Different letters on bars represent the significant values according to Duncan's test (*p* < 0.05).

The proline accumulation varies in different plant species and their organs under salinity stress. The proline content increased significantly in the leaves of all treated plants of *P. dactylifera* as the salinity increased (Figure 9). More accumulation of proline (2106.20 and 2632.99 *μ*g/g FW) was observed significantly under 100 and 150 mM NaCl treatments compared to control plants. Our findings related to proline accumulation were consistent with the results of Abdulwahid [72], who performed experiments on *P. dactylifera* under salinity stress. The proline was over accumulated in the roots and leaves of a date palm plant under abscisic acid, drought, and extreme temperatures and was remarkably high when leaves were exposed to suboptimum salinity and temperatures stresses [13]. The cultivars of *Phaseolus vulgaris* (Canario 60 and Pinto Villa) accumulated high proline content in leaves and shoots under 150 mM NaCl [74]. A high accumulation of proline was found under salinity stress in mulberry [75], green gram [76], *Jerusalem artichoke* [77], and canola [78]. The production of proline under stress conditions play an important role to protect the plant cells as it acts as soluble nitrogen sink, a signal of senescence, an osmoregulator, and an indicator of plant resistance [79].

**Figure 10:**
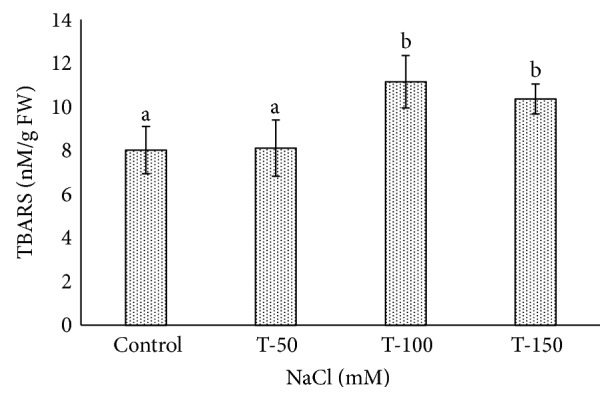
Effect of salinity stress on TBARS accumulation in *Phoenix dactylifera*. Data represent means of four replicates ± standard deviation. Different letters on bars represent the significant values according to Duncan's test (*p* < 0.05).

TBARS level increased in *P. dactylifera* plants under salinity stress as compared to control plants. A high content of TBARS (11.151 nM/g FW) was found in plants treated at 100 mM NaCl (Figure 10), and thereafter, a reduction was observed. However, result was found to be nonsignificant between control and plants treated at 50 mM NaCl. The plant species such as *Solanum nigrum* [80], *Artemisia annua* [81], *Glycyrrhiza uralensis* Fisch [82], and *Gypsophila aucheri* Boiss [83] showed increased TBARS content under salinity stress.

**Figure 11:**
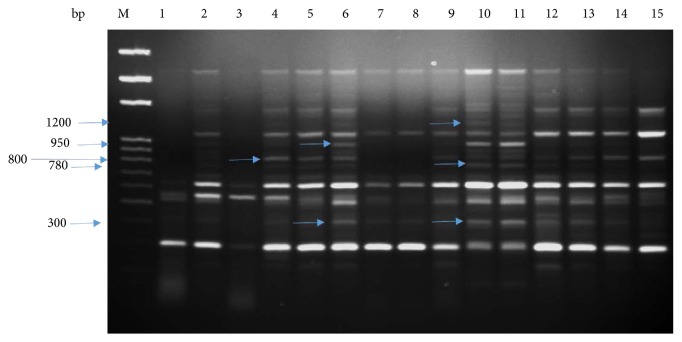
cDNA-SCoT marker profiling generated from individual plant leaf of *Phoenix dactylifera* at different concentrations of NaCl (SCoT primer 3). Lane M: 100 bp ladder; lanes 1, 2, and 3 (control); lanes 4, 5, 6, and 7 (50 mM NaCl); lanes 8, 9, 10, and 11 (100 mM NaCl); lanes 12, 13, 14, and 15 (150 mM NaCl).

**Figure 12:**
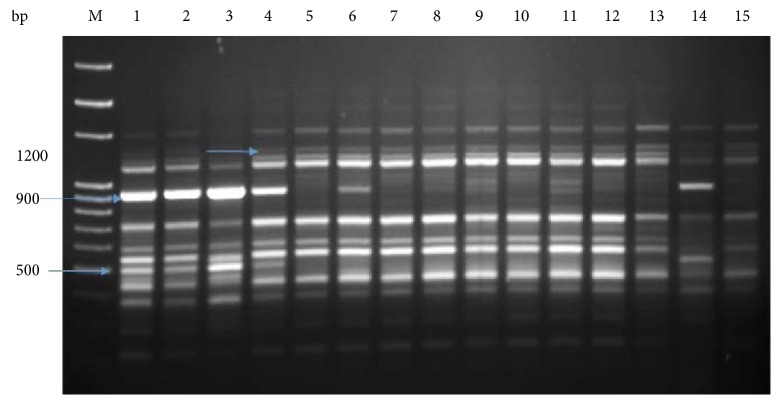
cDNA-SCoT marker profiling generated from individual plant leaf of *Phoenix dactylifera* at different concentrations of NaCl (SCoT primer 18). Lane M: 100 bp ladder; lanes 1, 2, and 3 (control); lanes 4, 5, 6, and 7 (50 mM NaCl); lanes 8, 9, 10, and 11 (100 mM NaCl); lanes 12, 13, 14, and 15 (150 mM NaCl).

**Figure 13:**
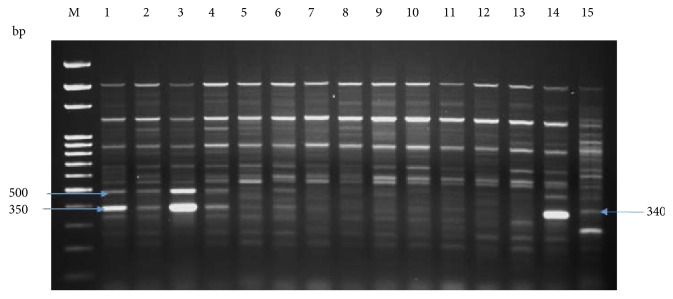
cDNA-SCoT marker profiling generated from individual plant leaf of *Phoenix dactylifera* at different concentrations of NaCl (SCoT primer 4). Lane M: 100 bp ladder; lanes 1, 2, and 3 (control); lanes 4, 5, 6, and 7 (50 mM NaCl); lanes 8, 9, 10, and 11 (100 mM NaCl); lanes 12, 13, 14, and 15 (150 mM NaCl).

We used cDNA-SCoT marker for the comparison of treated and untreated plants under salinity stress as the antioxidant system response and biomass of *P. dactylifera* were changed at various concentrations of NaCl stress which could be possible due to the expression of different genes. A single primer cDNA-SCoT technique has been applied to study gene expression in different plant species [43–45, 83–85]. The oligo-dT-anchored cDNA-SCoT was used in *M. indica* to study gene expression under abiotic stresses [44]. In our study, a different banding pattern was produced between treated and untreated plants using the cDNA-SCoT marker which indicated the expression of different genes under NaCl stress (Figures 11, 12, and 13). Different amplicons of size (1200, 950, 800, 780, and 300 bp) were produced in treated plants whereas were absent in control plants (Figure 11). The amplicon of size 950 bp was produced at 50 and 100 mM NaCl, whereas absent at 150 mM NaCl. Similarly, the amplicon size of 750 bp was produced at 100 mM NaCl and absent at 50 and 150 mM NaCl. The size of 1200 bp amplicon was produced in all treated plants, whereas 900 and 500 bp were produced in control as well as in plants treated at 50 mM NaCl (Figure 12). The size of amplicons 500 and 350 bp were produced in control as well as in plants treated at 50 mM NaCl whereas absent in plants treated at 100 mM NaCl (Figure 13). Similarly, the amplicon of size 340 bp was produced in plants treated at 150 mM NaCl (Figure 13). Thus, different NaCl concentrations impacted the expression profile of various genes which led to change in plant growth, biomass, and antioxidant system response. The cold resistance-related genes have been studied in sugarcane under cold stress using the cDNA-SCoT technique [84]. The differentially expressed genes in sugarcane, induced by *Leifsonia xyli* subsp. *xyli*, was studied using the cDNA-SCoT technique [85]. Wu et al. [43] used the cDNA-SCoT technique on sugarcane for the differential expression of gibberellin-induced genes for stalk elongation, which represented the upregulation and downregulation of genes.

Thus, based on the above results, *P. dactylifera* can be used by plant researchers to uncover the salt tolerant genes and their application in a plant-breeding program.

## Figures and Tables

**Table 1:** List of SCoT primer sequences used in the PCR reaction.

S.N.	Primer code	Primer sequence (5′-3′)
1	SCoT-1	CAACAATGGCTACCACCA
2	SCoT-2	CAACAATGGCTACCACCC
3	SCoT-3	CAACAATGGCTACCACCG
4	SCoT-4	CAACAATGGCTACCACCT
5	SCoT-5	CAACAATGGCTACCACGC
6	SCoT-6	CAACAATGGCTACCACGG
7	SCoT-7	CAACAATGGCTACCACGT
8	SCoT-8	CAACAATGGCTACCAGCA
9	SCoT-9	CAACAATGGCTACCAGCC
10	SCoT-10	AAGCAATGGCTACCACCA
11	SCoT-11	GCAACAATGGCTACCACC
12	SCoT-12	CATGGCTACCACCGGCCC
13	SCoT-13	ACCATGGCTACCACCGCA
14	SCoT-14	CCATGGCTACCACCGCAG
15	SCoT-15	ACCATGGCTACCACCGCA
16	SCoT-16	CCATGGCTACCACCGCAG
17	SCoT-17	CCATGGCTACCACCGCAC
18	SCoT-18	CCATGGCTACCACCGCCT
